# Spatiotemporal Variation in Rainfall Erosivity and Correlation with the ENSO on the Tibetan Plateau since 1971

**DOI:** 10.3390/ijerph182111054

**Published:** 2021-10-21

**Authors:** Bohao Cui, Yili Zhang, Linshan Liu, Zehua Xu, Zhaofeng Wang, Changjun Gu, Bo Wei, Dianqing Gong

**Affiliations:** 1 Key Laboratory of Land Surface Pattern and Simulation, Institute of Geographic Sciences and Natural Resources Research, CAS, Beijing 100101, China; cuibh.19b@igsnrr.ac.cn (B.C.); liuls@igsnrr.ac.cn (L.L.); wangzf@igsnrr.ac.cn (Z.W.); gucj.18b@igsnrr.ac.cn (C.G.); weib.20b@igsnrr.ac.cn (B.W.); gongdq.18s@igsnrr.ac.cn (D.G.); 2 College of Resources and Environment, University of Chinese Academy of Sciences, Beijing 100049, China; zhxu_st@rcees.ac.cn; 3 State Key Laboratory of Urban and Regional Ecology, Research Center for Eco-Environmental Sciences, Chinese Academy of Sciences, 18 Shuangqing Road, Beijing 100085, China

**Keywords:** rainfall erosivity, soil erosion, spatiotemporal variation, ENSO, Tibetan Plateau

## Abstract

Soil erosion is a serious ecological problem in the fragile ecological environment of the Tibetan Plateau (TP). Rainfall erosivity is one of the most important factors controlling soil erosion and is associated with the El Niño southern oscillation (ENSO). However, there is a lack of studies related to the spatial distribution and temporal trends of rainfall erosivity on the TP as a whole. Additionally, the understanding of the general influence of ENSO on rainfall erosivity across the TP remains to be developed. In this study, long-term (1971–2020) daily precipitation data from 91 meteorological stations were selected to calculate rainfall erosivity. The analysis combines co-kriging interpolation, Sen’s slope estimator, and the Mann–Kendall trend test to investigate the spatiotemporal patten of rainfall erosivity across the TP. The Oceanic Niño Index (ONI) and multivariate ENSO Index (MEI) were chosen as ENSO phenomenon characterization indices, and the relationship between ENSO and rainfall erosivity was explored by employing a continuous wavelet transform. The results showed that an increasing trend in annual rainfall erosivity was detected on the TP from 1971 to 2020. The seasonal and monthly rainfall erosivity was highly uneven, with the summer erosivity accounting for 60.36%. The heterogeneous spatial distribution of rainfall erosivity was observed with an increasing trend from southeast to northwest. At the regional level, rainfall erosivity in the southeastern TP was mainly featured by a slow increase, while in the northwest was more destabilizing and mostly showed no significant trend. The rainfall erosivity on the whole TP was relatively high during non-ENSO periods and relatively low during El Niño/La Niña periods. It is worth noting that rainfall erosivity in the northwest TP appears to be more serious during the La Niña event. Furthermore, there were obvious resonance cycles between the rainfall erosivity and ENSO in different regions of the plateau, but the cycles had pronounced discrepancies in the occurrence time, direction of action and intensity. These findings contribute to providing references for soil erosion control on the TP and the formulation of future soil conservation strategies.

## 1. Introduction

Soil erosion has already emerged as one of the most serious ecological and environmental problems globally, which not only threatens terrestrial ecosystems, but also severely restricts the security of human existence and the sustainable development of economy and society [1,2]. Soil erosion not only contributes to land degradation, but even interferes with the ability of the soil carbon cycle to mitigate the greenhouse effect [3,4]. Soil erosion by water is considered to be one of the most detrimental types of soil erosion, causing a loss of soil nutrients, which reduces crop yields, pollutes water quality, contributes to the sedimentation of rivers, and raises flooding [5,6,7,8,9]. Therefore, the accurate prediction of water erosion is of great significance for the comprehensive management of soil erosion and effective soil protection.

The causes of water erosion are related to a series of natural factors involving rainfall, soil, topography, vegetation, and other human factors such as land use and crop cultivation management [10,11]. In particular, rainfall is the principal climatic factor responsible for water erosion, which influences water erosion through the duration, amount, and intensity of rainfall events [12]. The principal predictive tools for water erosion are the Universal Soil Loss Equation (USLE) and the Revised Universal Soil Loss Equation (RUSLE), which have been applied worldwide, and rainfall erosivity (R-factor), one of the key input parameters in the model, is the potential capacity of rainfall to induce water erosion [12,13]. The R-factor is defined as the product of the rainfall energy and the maximum rainfall intensity in a 30 min period (EI30), and the calculation requires the use of consecutive rainfall data series with a temporal resolution of at least 15 min, which is however, hardly available in many countries and regions. Even if an adequate rain gauge data can be accessed, the complicated calculation process is time-consuming and laborious, which dramatically restricts model promotion and implementation [14].

In this context, as alternative algorithms based on the relationship between R-factor and available rainfall data were developed, including the calculation of rainfall erosivity based on annual [15,16], monthly [17,18], and daily [19,20] rainfall data from meteorological stations or satellite radar [21,22]. Among these, daily rainfall data are widely used due to their relative accessibility, which provides more characteristic information of rainfall and facilitates the precision and reliability of R-factor estimation [23]. In the daily rainfall data model, the rainfall erosivity algorithm was divided into linear exponential, logarithmic, and power functions to fit the relationship between rainfall and rainfall erosivity, and the models are mostly combined experimental and empirical based [24,25,26]. In general, these models perform an important role in the quantitative evaluation of rainfall erosivity, and provide scientific reference for the forming mechanism of water erosion, evolutionary process and even the mechanics of climate change.

It has become an indisputable fact that the global climate is changing remarkably, with extreme weather events growing stronger, more frequent, and lasting longer [27]. El Niño southern oscillation (ENSO) is the most intense sea-air interaction event affecting the global climate, and although it usually occurs in the eastern equatorial Pacific region, it can be responsible for rainfall anomalies spreading globally [28]. The ENSO cycle has a pronounced periodic character as a result of the interaction between the ocean and the atmosphere, with El Niño (warm phase) and La Niña (cold phase) as the two extreme phases of the ENSO cycle. Considerable work has been conducted on the relationship between ENSO and precipitation events, anomalous temperature, wet and dry variability, and atmospheric circulation [29,30,31,32]. The studies also pointed out that El Niño and La Niña showed diverse rainfall patterns, for example, compared with the La Niña period, northern China is more arid during El Niño in the northern hemisphere, while rainfall in the southeast of China appears to increase substantially, while the contrary phenomenon is present in the southern hemisphere [33,34]. Although these studies have enhanced our comprehension of atmospheric tele-correlation model (ENSO) effects on rainfall, currently the effect of ENSO on rainfall erosivity is still only shown in a few studies [35,36,37,38,39]. A significant dependence between rainfall erosivity and the ENSO indices has been observed in eastern China [35,37], northeastern Spain [40], and the southwestern United States [41], while studies on how ENSO affects rainfall erosivity on the TP are still unknown. 

The Tibetan Plateau (TP) is the largest and highest geographical unit in the world, with an average altitude of over 4000 m, and is called the Earth’s “third pole”. It is of extreme importance to regional economic development and ecological security, as well as global climate, water resources, and ecosystem functioning [42]. Since the 21st century, however, drastic environmental changes have been remarkably observed on the TP [43]. These changes have become key drivers of increased soil erosion risk. Studies have demonstrated that grassland ecosystems on the TP are suffering from severe degradation due to the combined effects of climate change and human activities. This in turn has triggered a decline in biomass, biodiversity, and landscape complexity, fragmentation or complete loss of services such as soil and water conservation, and an increase in rainfall erosivity and sandstorms [44,45,46,47]. Permafrost degradation can reduce the stability of soil aggregates and the water content in the surface soil is abnormally high during the thawing stage, thus shortening the time of runoff generation and exacerbating erosion caused by rainfall [48,49]. The glaciers’ retreat and the rise of the snow line in cold areas at high altitudes have changed the surface albedo and atmospheric heat circulation and thus have affected the local rainfall intensity, and the form of erosion caused by glacial meltwater and snowmelt runoff generated is one of the main reasons for increased erosion [50,51]. Additionally, according to observations and climatological models, the TP has suffered a faster rate of warming since the 1960s, which is three times the global average [52]. Notable changes in the plateau climate system, such as short periods of intense rainfall triggered by extreme precipitation events, may have led to an increasing trend in the rainfall erosivity on the TP [51]. Some studies have analyzed the variation in rainfall erosivity in the catchment and local scales of the TP, indicating an increasing trend of rainfall erosivity [53,54,55]. These studies provided useful information on the variation in rainfall erosivity, but a further analysis is necessary for the TP as a whole.

Soil erosion is serious on the TP, with 70% of the area suffering from varying degrees of soil erosion [56]. In both sides of the Yarlung Tsangpo River and the South Qiangtang area, gully erosion is widely distributed, while in the interior of the plateau scale erosion becomes the main type of erosion in grasslands [57]. Soil erosion on the TP has caused irreparable soil degradation and land area reduction, and is leading to the sedimentation of downstream rivers, landslides, mudslides and other disasters, posing a threat to transportation, agriculture, and animal husbandry. Moreover, soil conservation is particularly important in the TP due to its harsh physical environment, widespread permafrost and fragile alpine ecosystems making it the most sensitive and fragile region [58]. Once erosion happens, its rehabilitation process is prolonged and difficult.

Detection of long-term trends in rainfall erosivity can provide information regarding the potential impact of rainfall changes on soil erosion. It is particularly useful for the TP region, which is more sensitive to water erosion and climate change because of the fragile biophysical conditions [59]. However, these unique geographical features and complicated terrain have restricted soil erosion studies due to the scarce observational data on precipitation and soil erosion. Previous studies have focused on local watersheds or small areas of the TP, while the spatial and temporal characteristics of how rainfall erosivity vary over the entire TP have not been adequately studied [60,61]. Moreover, periodic factors lead to ‘poverty years’ and ‘abundant years’ of precipitation in the highlands in different years. The interannual variation in precipitation erosivity on the TP may be the result of ENSO action, but the general effect of ENSO on rainfall erosivity in the TP is not clear at present, and it is necessary to expand the related understanding.

In view of this, the TP as a whole was chosen as the study area, and daily rainfall data from 91 meteorological stations were selected to calculate the rainfall erosivity and to explore its relationship with ENSO. The objectives of the study are as follows: (1) to characterize the temporal trends of rainfall erosivity during 1971–2020 across the entire TP; (2) to present the spatial distribution of rainfall erosivity on the TP; (3) and to investigate the impacts of ENSO on rainfall erosivity in different regions of the TP.

## 2. Materials and Methods

This study was based on a single case study of the TP. In this section, the basic information about the study area, the required data handling process and the methods related to rainfall erosivity were described in detail.

### 2.1. Study Area

**Figure 1:**
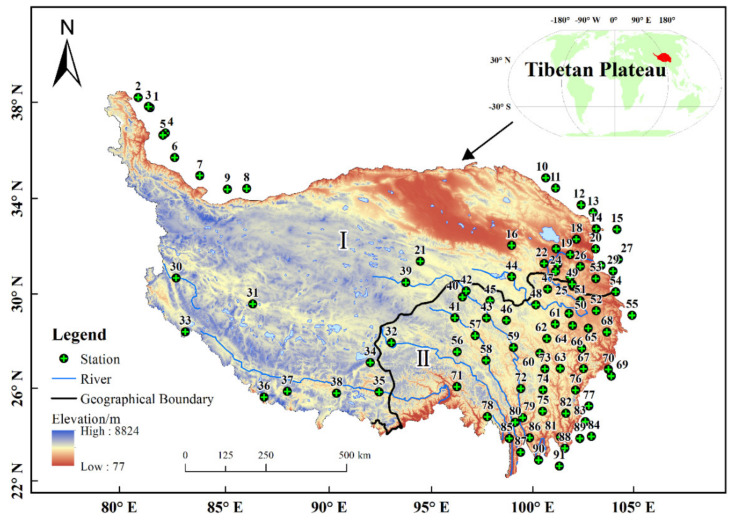
Study area of Tibetan Plateau (TP) and the distribution of meteorological stations.

The Tibetan Plateau is located in the southwestern part of China, with an area of 2.74 × 10^6^ km^2^ and an average altitude of over 4000 m. It is known as the “roof of the world” [62]. It is included in the Tibet Autonomous Region and Qinghai Province, and the southern part of the Xinjiang Uygur Autonomous Region, the western part of Gansu Province, the western part of Sichuan Province and the northern part of Yunnan Province. The main mountain ranges are the Kunlun Mountains, Qilian Mountains, Karakorum Mountains, Himalayas, and Hengduan Mountains. The climate ranges from a humid monsoon climate in the southeast to an alpine arid plateau climate in the northwest, controlled by the Pacific monsoon, Indian monsoon, and prevailing westerly winds, and is influenced by the mountain terrain [63]. Diverse climate types form subtropical rainforests, shrubs, alpine meadows, alpine grasslands, and alpine desert vegetation types are present. TP precipitation exhibits a distinct gradient, gradually decreasing from more than 1000 mm in the southeast to less than 50 mm in the northwest [64]. The region has experienced soil erosion, desertification and landslide hazard [51,65]. Referring to [66], the criteria for the physical geographic zoning of the TP divided the plateau into Region I (arid zone) and Region II (humid zone) (Figure 1).

### 2.2. Database

#### 2.2.1. Daily Rainfall Data

The observed daily precipitation data used in this study was obtained from the Climatic Data Center, National Meteorological Information Center of the China Meteorological Administration (CMA) (http://data.cma.cn (accessed on 10 October 2021)). The data included a total of 91 meteorological stations (Figure 1), with complete data series, covering the time period 1971 to 2020 (Table S1). Moreover, considering the continuity of spatial interpolation and the stations spreading over the entire TP as much as possible, 27 meteorological stations around the study area were selected with the criterion that the shortest linear distance from a meteorological station to the TP boundary is not greater than 100 km. The observation records of all surrounding stations were recorded at the same time as the study period. In order to ensure data reliability and continuity, each meteorological data record was evaluated by the National Meteorological Center [67].

#### 2.2.2. ENSO Indices

ENSO is a phenomenon of irregular periodic changes in sea surface temperature and wind occurring in the equatorial eastern Pacific Ocean, one of the strongest natural signals of interannual climate change worldwide. The typical characteristics of ENSO events are commonly known to be anomalous SSTs (±0.5 °C) in the eastern Pacific Ocean for more than 5 months, where warm episodes are El Niño events and cold episodes are La Niña events [68,69]. The multivariate ENSO Index (MEI) was obtained as the first non-rotating principal component (PC) of the six variables (sea-level pressure, zonal and meridional components of the surface wind, sea surface temperature, surface air temperature, total cloudiness fraction of the sky) over the tropical Pacific [70,71]. it is considered as a better index for detecting the ENSO phenomena with respect to other indices because it takes into account more information and fewer data failures [37]. Therefore, in this study, the occurrence and duration of the El Niño event and La Niña event were determined based on the Oceanic Niño Index (ONI), and MEI was selected as the ENSO proxy to probe the relationship between rainfall erosivity and ENSO during the time period of 1971–2020. These indexes are obtained from the National Oceanic and Atmospheric Administration (NOAA). Specifically, ONI was acquired from NOAA Climate Prediction Center [72], and MEI was acquired from NOAA Earth System Research Laboratory.

### 2.3. Methods

#### 2.3.1. Technical Route

**Figure 2:**
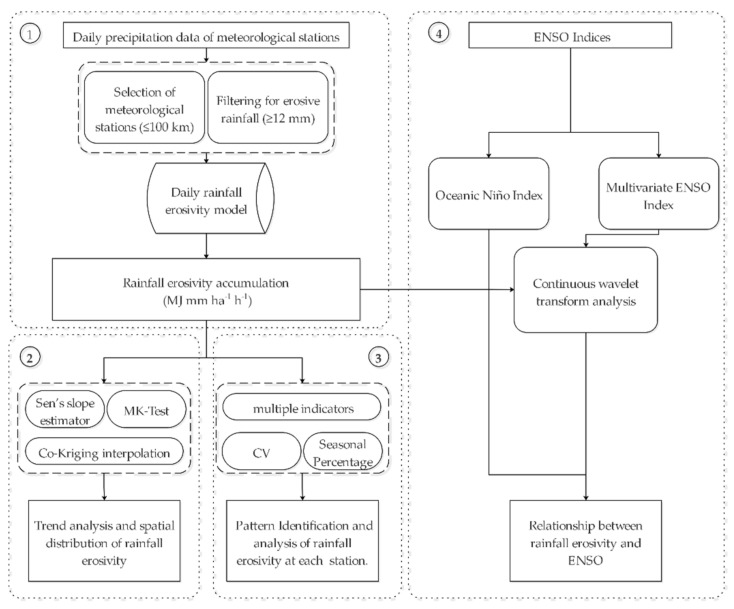
Technical route. Note: MK-test refers to Mann–Kendall test, CV refers to the coefficient of variation.

The study was divided into four steps (Figure 2):

Step 1: Estimation of rainfall erosivity at different time scales. Based on the daily rainfall data of 91 stations, the annual, seasonal, and monthly average rainfall erosivity of TP from 1971 to 2020 were calculated using the daily rainfall erosivity model.

Step 2: Trend analysis and spatial distribution of rainfall erosivity in TP. Firstly, the temporal changes trend of rainfall erosivity was evaluated by using Sen’s slope estimation and the MK trend test, and then the co-kriging method was used for the spatial mapping of rainfall erosivity for the period of 1971–2020. 

Step 3: Pattern identification and analysis of rainfall erosivity change at each meteorological station. Firstly, the change trend of rainfall erosivity at each station was classified by integrating multiple indicators. Then, the coefficient of variation (CV) and the seasonal spatial distribution of rainfall erosivity of each site was analyzed.

Step 4: Relationship between rainfall erosivity and ENSO. Based on ONI, the variation in monthly mean rainfall erosivity during El Niño and La Niña events from 1971 to 2020 was analyzed; based on the MEI index, a continuous wavelet transform analysis method was used to examine the influence of ENSO on rainfall erosivity, and to clarify the response of the resonance period in different regions of the TP.

#### 2.3.2. Calculation of Rainfall Erosivity

The half-monthly rainfall erosivity was estimated for each of the 91 meteorological stations from 1971 to 2020 using the daily rainfall erosivity model. The agent model was originally built from Richardson’s equation [73], and later improved by Zhang [20]. Previous studies have demonstrated that this method is reliable and has been widely used on the national and regional scales in China [36,74,75,76]. This method is based on daily rainfall data to obtain monthly, seasonal, and annual rainfall erosivity. The calculation procedures are as follows:

(1) $$R_{i}=\alpha \sum_{j=1}^{k}{\left(P_{j}\right)}^{\beta }$$

(2) $$\alpha =21.586\beta^{-7.1981}$$

(3) $$\beta =0.8363+\frac{18.144}{P_{\left(d12\right)}}+\frac{24.455}{P_{\left(y12\right)}}$$

where $R_{i}$ is the rainfall erosivity in the *i-*th half-month period (MJ mm ha^−1^ h^−1^), *k* is the number of days in the half-month period, and $P_{j}$ is the daily erosive rainfall amount (mm) on the *j*-th day during the half-month period. The half-month interval method is as follows: with the fifteenth day of each month as the dividing point, the whole year is divided into 24 half months. The half-month period as a basic statistical unit is used to calculate the corresponding half-month rainfall erosivity. 

According to the national rainfall and runoff analysis, ≥12 mm is defined as erosive rainfall [77]. Therefore, the daily rainfall ≥12 mm is applied to Formula (1), otherwise, it is regarded as value of 0 in the calculations.

The terms α and β are two parameters to be determined in the model. $P_{\left(d12\right)}$ is the average daily erosive rainfall amount (mm) and $P_{\left(y12\right)}$ is the average annual erosive rainfall amount (mm). In this study, Formulas (1)–(3) are used to calculate the half-month rainfall erosivity of each meteorological station. The annual and seasonal rainfall erosivity of is the cumulative value of rainfall erosivity in every half-month period.

#### 2.3.3. Sen’s Slope Estimator and Mann–Kendall Test

In this study, the trends magnitude of annual rainfall erosivity was estimated with the non-parametric Sen’s method. The trends and significance of annual and seasonal (monthly) rainfall erosivity were detected with the non-parametric Mann–Kendall test. 

Sen’s slope estimation is a non-parametric method of slope calculation, which is commonly used in the trend analysis due to its high robustness and computational efficiency [78]. The determination for the slope of annual rainfall erosivity is as follows: first, the values of $Q_{i}$ calculated by the Formula (4) are ranked in order of magnitude, and then determines the overall estimator ($SLOPE_{med}$) as the median of these $Q_{i}$ by Formula (5).

The slope in the *N* pairs of samples is calculated as follows:

(4) $$Q_{i}=\frac{x_{j}-x_{k}}{j-k}\;\left(i=1,\ldots ,N\right)$$

where $x_{j}$ and $x_{k}$ are values of the rainfall erosivity corresponding to periods *j* and *k*, respectively (*j* > *k*). $SLOPE_{med}$ is calculated according to the following formula:

(5) $$SLOPE_{med}=\left\{\begin{array}{ll} Q_{\left[\frac{N+1}{2}\right]}<0 & \mathrm{if}\;N\;is\;odd\;\\ \frac{Q_{\left[\frac{N+1}{2}\right]}+Q_{\left[\frac{N+1}{2}\right]}}{2} & \mathrm{if}\;N\;is\;even \end{array}\right.$$

where $SLOPE_{med}$ > 0 indicates an upward trend, and vice versa. Its value indicates the magnitude of the trend change.

The non-parametric Mann–Kendall test is a widely used technique for to assess the significance of trends in long time series [79,80]. It is distribution free and not affected by missing values and outliers, and is highly recommended by the World Meteorological Organization [81]. This method is primarily based on two parameters, *S* and *Z*, to determine whether a time series has a significant trend. The intermediate variable *S* is computed as:

(6) $$S=\overset{n-1}{\underset{k=1}{\sum }}\overset{n}{\underset{j=k+1}{\sum }}\;\mathrm{sgn}\left(x_{j}-x_{k}\right)$$

where *n* is the length of the time series, $x_{j}$ and $x_{k}$ are values of the rainfall erosivity corresponding to periods *j* and *k*, respectively (*j* > *k*). S is the summation of $\mathrm{sgn}\left(x_{j}-x_{k}\right)$, which takes the value of −1, 0, or 1 when $\left(x_{j}-x_{k}\right)$ is less than, equal to, or greater than 0, respectively. The variance of *S* can be acquired as follows:

(7) $$var\left(S\right)=\frac{n\left(n-1\right)\left(2n+5\right)}{18}$$

Then the normalized statistical value Z is denoted as follows:

(8) $$Z=\left\{\begin{array}{ll} \frac{S-1}{\sqrt{var\left(S\right)}} & \mathrm{if}\;S>0\\ 0 & \mathrm{if}\;S=0\\ \frac{S+1}{\sqrt{var\left(S\right)}} & \mathrm{if}\;S<0 \end{array}\right.$$

where a positive (negative) value of Z indicates an upward (downward) trend. In bilateral trend detection, a time series with a significant trend is indicated if *|Z|≥ Z*_1−*α*/2_ at a certain significance level *α*, where *Z*_1−*α*/2_ is obtained from the standard normal cumulative distribution tables. The trend is statistically significant at the 0.1, 0.05, and 0.01 significance level when *|Z|* > 1.645, 1.96 and 2.576, respectively. Besides, the Mann–Kendall test can also be used to detect the abrupt points. The abrupt points and the approximate time of occurrence can be located according to the intersection of the progressive and retrograde sequences within the sequence. More details of the abrupt points calculation on the Mann–Kendall Test are available from the network resources. 

#### 2.3.4. Spatial and Statistical Analysis

The mean annual rainfall erosivity at each of the 91 stations was calculated by a long-term (1971–2020) average value of annual rainfall erosivity. Based on these station’s values, the co-kriging interpolation method was used to interpolate the spatial distribution of the average annual erosivity of the TP, using the geostatistical analysis tool ArcGIS 10.4.

Different from the inverse distance weighting (IDW) method which only considered one assumption: nearby points should be closer to the value of the interpolation position than distant points, the co-kriging interpolation method allowed the addition of covariates to improve the accuracy of estimation or prediction [74,82]. Considering the complex terrain of the TP, the elevation factor was defined as a co-variable in the co-kriging interpolation method [36]. The elevation data of each meteorological station was provided by the China Meteorological Administration (CMA). Based on the values of rainfall erosivity for 91 meteorological stations on the TP, the co-kriging interpolation method was performed and generated the spatial distribution map of rainfall erosivity for the period of 1971–2020.

In this study, the rainfall erosivity anomalies of the TP is expressed as the difference between the annual erosivity value of the observation year and the 50-year average value. The 5-year moving average anomaly can smooth fluctuations and reduce potential errors, and was used to analyze the temporal changes of the rainfall erosivity across the TP. The seasonal (monthly) average rainfall erosivity of the TP was calculated by averaging the seasonal (monthly) erosivity of 91 meteorological stations during the same time span, from 1971 to 2020. The annual variation from the meteorological site is represented by the coefficient of variation (CV), which is expressed as a percentage of the standard deviation of the annual erosivity to the average of the observation year. The map of seasonal spatial distribution of rainfall erosivity is expressed as a percentage of the seasonal rainfall erosivity in the annual total erosivity at each weather station. 

#### 2.3.5. Identification of Rainfall Erosivity Trend Patterns

With reference to the time series trend identification method of Ray [83], rainfall erosivity change patterns were identified for each meteorological station from 1971 to 2020. Multiple indicators were integrated: the *Z* values calculated by Mann–Kendall, *SLOPE%* and *R_ST_*.

The *SLOPE*% value represents as a percentage of *SLOPE* for the average rainfall erosivity for each meteorological station during 1971–2020. The calculation formula is as follows:

(9) $$SLOPE\%=\frac{SLOPE}{\left(\sum_{i=1}^{n}\;x_{i}\right)/n}\times 100$$

where *SLOPE* is the Sen’s slope value of rainfall erosivity changes, $x_{i}$ is the value of the rainfall erosivity corresponding to period i, and *n* is the length of the time series. The *R_ST_* is defined as the ratio of the average rainfall erosivity for the last 3 years to the maximum 3 year moving average. This is used to identify whether the increasing trend of annual rainfall erosivity is interrupted, shifting to a decline at later stages. The equation is expressed below:

(10) $$R_{ST}=\frac{\mathrm{ave}\left(x_{n-2},x_{n-1},x_{n}\right)}{max\left(A\mathrm{VE}\left(x_{1},x_{2},x_{3}\right),\mathrm{AVE}\left(x_{2},x_{3},x_{4}\right),\ldots AVE\left(x_{n-2},x_{n-1},x_{n}\right)\right)}$$

where $x_{i}$ is the value of the rainfall erosivity corresponding to period i, and *n* is the length of the time series.

The trend of rainfall erosivity was classified by the above-mentioned three indicators into four patterns of decreasing, stagnant, increasing-stagnant and increasing (Table 1), abbreviated as DE, ST, IN-ST and IN, respectively. The *Z* value indicates whether there is a significant trend of rainfall erosivity (|*Z*| > 1.96 at the 0.05 significance level), a non-significant change trend (0.675 < |*Z*| ≤ 1.96 at the 0.05–0.5 significance level), and no change trend (|*Z*| ≤ 0.675 at the below 0.5 significance level); *SLOPE*% indicates whether the magnitude of the rainfall erosivity trend change is significant. References [84,85] used 0.25% as a criterion, i.e., a change greater than 0.25% is assumed to be significant. $R_{ST}$ is used to determine whether the annual rainfall erosivity growth trend is interrupted, or turns down and mitigates at a later stage.

#### 2.3.6. Continuous Wavelet Transform Analysis

The continuous wavelet transform (CWT) is a method to decompose a time series into a two-dimensional phase plane of the time-frequency simultaneously. It is commonly applied to the analysis of various hydrological and meteorological processes with high variability to detect non-stationary trends, periodicities, and durations as it better characterizes oscillatory behaviors of signals than discrete wavelet transforms [86,87,88]. Specifically, two CWTs, cross wavelet transform (XWT), and wavelet transform coherence (WTC), were constructed to investigate whether there is any periodicities or correlations between rainfall erosivity and ENSO. The XWT reveals regions of high common power in the time-frequency spectrum, and calculates the phase relationships between signals. The WTC identifies two time series variation correlations in both time and frequency space, even in the absence of high-power regions. In this study, the wavelet power spectrum of CWT was employed to analyze the relationship and the possible periodicity between rainfall erosivity in different regions and changing patterns of MEI. XWT revealed high common power regions and phase relationships between the two variables, and WTC was used to determine the correlation position of the two variables at local scales. The CWT toolbox package for MATLAB was used to perform all wavelet analyses. For further details about CWT, refer to [89].

## 3. Results

In the following section, the results are represented according to the technical approach mentioned in Section 2. This section analyzed the variability characteristics of rainfall erosivity at different time scales and the spatial distribution pattern of rainfall erosivity at each station, while identifying the relationship between rainfall erosivity and ENSO on the TP.

### 3.1. Variation Characteristics of Annual Rainfall Erosivity

**Figure 3:**
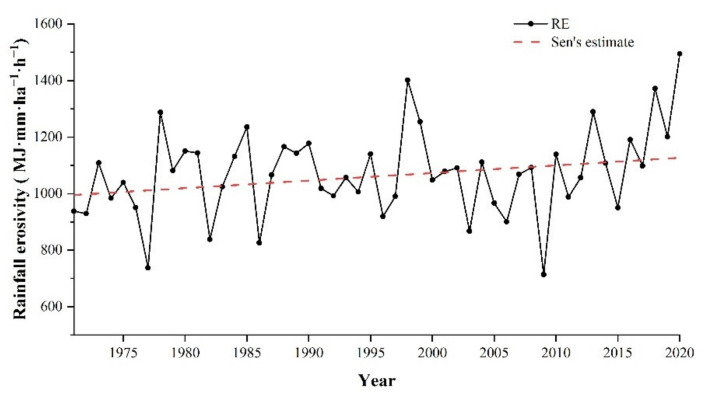
Annual variation in rainfall erosivity on the TP from 1971 to 2020, the red dotted line represents Sen’s estimate.

Sen’s slope estimation analysis showed an increasing trend of rainfall erosivity on the Tibetan Plateau from 1971 to 2020, with a Sen’s slope value of 2.69 for annual rainfall erosivity (Figure 3). The average annual erosivity range from 713.50 to 1495.41 MJ·mm·ha^−1^·h^−1^, with a multi-year average erosivity of 1071.42 MJ·mm·ha^−1^·h^−1^. An anomaly analysis indicated obvious inter-annual fluctuation in rainfall erosivity. The magnitude of rainfall erosivity undulation was relatively small until 1996 and increased significantly after 1996, with longer fluctuation periods. For the entire study period, the annual rainfall erosivity was above the mean for the same duration as the periods below the mean, with the highest value of 1495.41 MJ·mm·ha^−1^·h^−1^ in 2020; the lowest value of 713.50 MJ·mm·ha^−1^·h^−1^ in 2009; the extreme value ratio was 2.1 (Figure S1). Meanwhile, the Mann–Kendall trend analysis had a Z value of 1.67, indicating that this trend passed the significance test at the 90% confidence level, with the mutation point occurring in approximately 2017 (Figure S2).

### 3.2. Changes in Seasonal and Monthly Rainfall Erosivity

**Figure 4:**
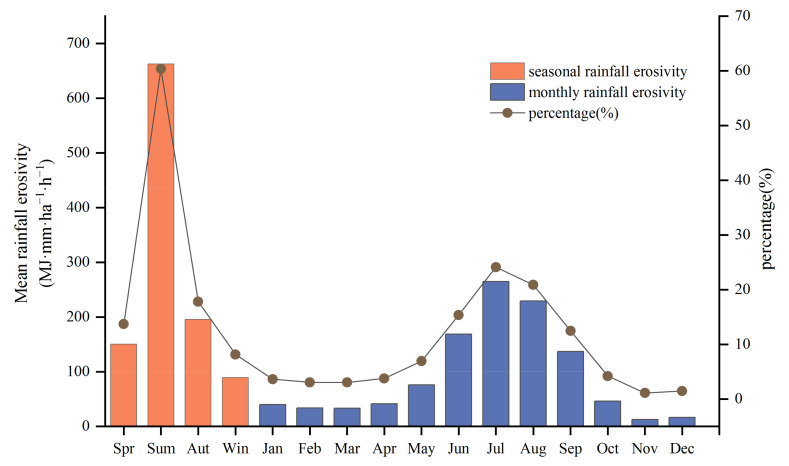
Statistics of seasonal and monthly average rainfall erosivity and its percentage.

The seasonal mean rainfall erosivity showed significant discrepancies at 91 meteorological stations on the TP. The rainfall erosivity in order was summer > autumn > spring > winter, with a range of 89.33–662.58 MJ·mm·ha^−1^·h^−1^. In particular, the average rainfall erosivity in summer was the highest, accounting for 60.36%, while winter was the lowest, accounting for only 8.14% of the total annual erosivity (Figure 4). This phenomenon was mainly influenced by the heterogeneity of the seasonal distribution of precipitation. With the transport of water vapor from the North Indian and Western Pacific monsoons, the summer monsoon brought 58.5% of the year’s rainfall, while late spring and early autumn accounted for 90% of the year’s rainfall [90]. As shown in Figure S3, the summer rainfall erosivity showed a non-significant increasing trend, with the MK statistical value of 1.54. In contrast, there was a decreasing trend in spring, autumn, and winter rainfall erosion; the spring and autumn MK statistic passed the significance test (*p* = 0.05), which were −2.19 and −2.09, respectively.

Although the monthly average rainfall erosivity was highly variable, there was a clear temporal consistency with the seasonal rainfall erosivity. June, July, and August, corresponding to summer, were the three months with the highest percentage of rainfall erosivity for the year. The monthly rainfall erosivity was 168.73 MJ·mm·ha^−1^·h^−1^, 264.75, and 229.10 MJ·mm·ha^−1^·h^−1^, respectively. Rainfall erosivity was highest on the Tibetan Plateau in July, the proportion of erosivity reached 24.19% for the year. November was the lowest with 1.12%, but a higher variability was found in this period, with a high extreme ratio of 18.71 (Figure 4). The trends in the monthly average rainfall erosivity from 1971 to 2020 were further examined, showing an increasing trend in eight months and a decreasing trend in four months. Specifically, the greatest increasing trend in mean rainfall erosivity was in August, but the increasing trend was insignificant in all months. Three months showed a significant decreasing trend, including March and April at 90% and 95% confidence levels, respectively, and the most significant decreasing trend was in November, which passed 99% confidence level (Figure S3).

### 3.3. Spatial Patten of Rainfall Erosivity in the Tibetan Plateau

**Figure 5:**
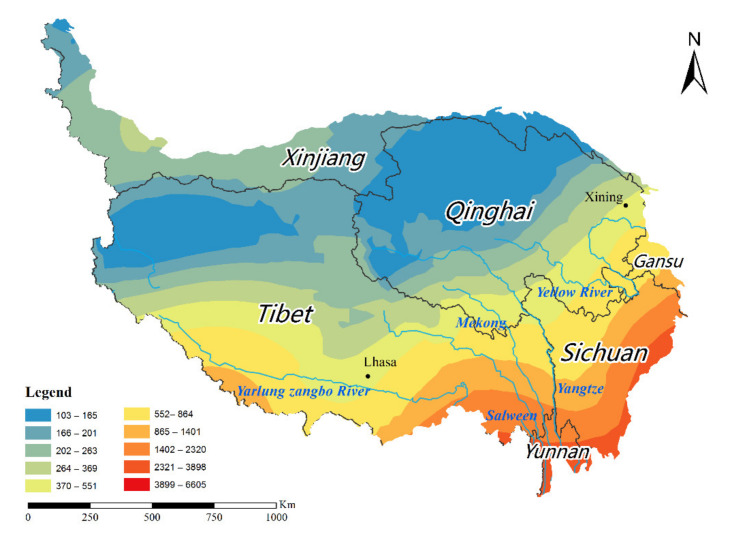
Spatial distribution of rainfall erosivity on the TP during the period from 1971 to 2020.

In order to reduce the boundary effect on the annual rainfall erosivity spatial pattern, 91 meteorological stations were selected for the interpolation better to reveal the spatial variation in rainfall erosivity. In general, the rainfall erosivity on the TP from 1971 to 2020 had obvious spatial differences, roughly exhibiting a spatial pattern of decreasing distribution from southeast to northwest (Figure 5). The high value zone of rainfall erosivity was approximately in the southeastern part of the TP, mainly distributed in the lower altitude regions such as the Hengduan Mountains and the Yarlung Tsangpo Valley. There were three stations with an average annual rainfall erosivity greater than 6000 MJ·mm·ha^−1^·h^−1^, of which the highest value occurs at Dujiangyan station in Sichuan Province, with an average annual rainfall erosivity of 6605.21 MJ·mm·ha^−1^·h^−1^. The other two stations are Gongshan station and Huaping station in Yunnan Province, with an average annual rainfall erosivity of 6152.44 MJ·mm·ha^−1^·h^−1^ and 6193.75 MJ·mm·ha^−1^·h^−1^, respectively. The zones with low average annual rainfall erosivity were mainly found in the northern and western parts of the TP, including concentrations in the Qiangtang Plateau and the Qaidam Basin. For example, the lowest value was at Shiquanhe station in the Tibet Autonomous Region, where the annual rainfall erosivity was only 103.46 MJ·mm·ha^−1^·h^−1^. In summary, the average annual rainfall erosivity was less than 500 MJ·mm·ha^−1^·h^−1^ which accounted for 48.35% of all stations, 500–1000 MJ·mm·ha^−1^·h^−1^ for 24.16% and that of more than 1000 MJ·mm·ha^−1^·h^−1^ accounted for 27.47%.

The long time series trend analysis of each meteorological station on the TP from 1971 to 2020 showed that the annual rainfall erosivity exhibited an increasing trend at 49 meteorological stations, accounting for 54% of all stations. Thereby this demonstrated the reason for the increasing rainfall erosivity across the entire TP since 1971 (Figure S4). Of these, 13 meteorological stations increased consistently, mainly in the eastern Hengduan Mountains of the TP, accounting for 27% of the total increases. The meteorological stations that showed a pattern of rainfall erosivity increased first and then gradually stabilized were mainly located in the Hehuang valley in the northeastern part of the Tibetan Plateau and the southern Tibetan valley in the southeast, accounting for 27% of the total increase in stations. The annual rainfall erosivity showed a long-term stable (no significant trend) pattern at 37 meteorological stations, accounting for 41% of all meteorological stations. Only five stations showed a gradual decrease in annual rainfall erosivity, accounting for 5% of all meteorological stations, but both patterns of change were not significant. In addition, as shown in Table S2, further analysis based on the statistics results of the multiple indicators at each station revealed that the types of trends at each site differ significantly in terms of significance levels and magnitude of change. Of all the increasing pattern stations, the significant increases were seen at Min station (*p* = 0.05) and Derong station (*p* = 0.01). The largest and smallest increases were at Min station and Pishan station, respectively. Of all the increasing-stagnating pattern stations, there are five meteorological stations at more than 95% confidence level, namely Zekog station (*p* = 0.01), Gerze station (*p* = 0.01), Dulan station (*p* = 0.01), Wuwei station (*p* = 0.01), and Guinan station (*p* = 0.05). Of all the decreasing pattern stations, the largest and smallest increases were at Gongshan station and Artux station, respectively.

**Figure 6:**
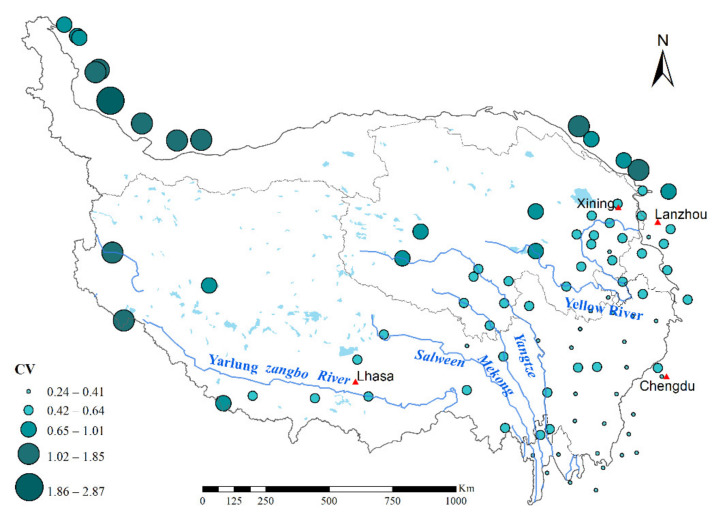
Spatial distribution of the coefficient of variation (CV) in rainfall erosivity during 1971–2020.

As shown in Figure 6, the mean coefficient of variation (CV, the ratio of the standard deviation to the mean) in the interannual rainfall erosivity for 91 stations since 1971 was 0.61, indicating moderately high rainfall erosivity variability across the plateau. The spatial distribution pattern of CV had high consistency with annual rainfall erosivity. In other words, the CV increased from south-east to north-west. Specifically, 11 meteorological stations were in regions of intense variation (CV > 1), mostly in the north-western flank of the Kunlun Mountains on the TP, the Ali Mountains in the Tibet Autonomous Region, and the northern Qilian Mountains in eastern Qinghai Province. while 54 meteorological stations were in regions of lesser variation (CV < 0.5), accounting for 59% of the total meteorological stations, mainly in the south-eastern part of the TP. Furthermore, the meteorological station with the smallest CV in the interannual rainfall erosivity was Jiulong Station, located in Sichuan Province in the southeastern part of the Tibetan Plateau, while the largest CV was Pishan Station, located in Xinjiang Autonomous Region in the northwestern part of the Tibetan Plateau. In summary, over the past half century, rainfall erosivity exhibited clear spatial disparities on the TP, specifically, annual rainfall erosivity in the southeast were mainly characterized by a slowly and steadily increase, while annual rainfall erosivity in the northwestern part of the plateau showed greater fluctuations and instability, with no significant trends.

**Figure 7:**
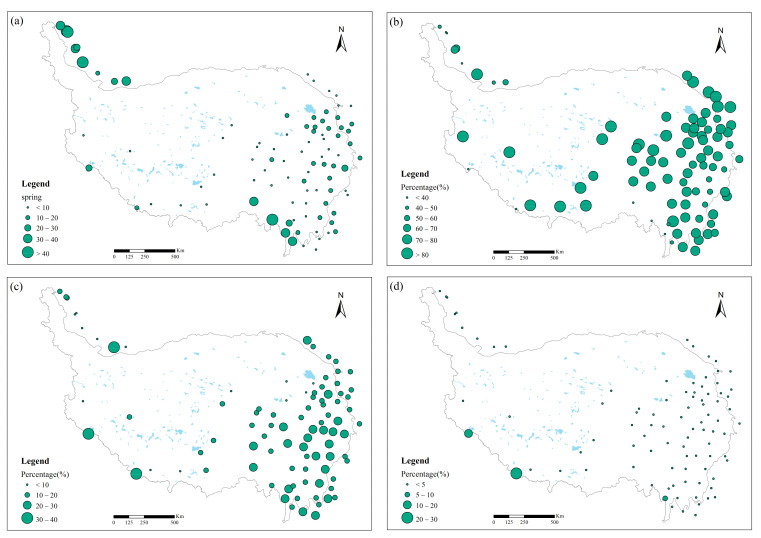
Spatial pattern of rainfall erosivity from 1971 to 2020 in spring (**a**), summer (**b**), autumn (**c**), and winter (**d**). Note: Percentage refers to the proportion of seasonal rainfall erosivity to total annual erosivity.

The seasonal spatial distribution pattern of rainfall erosivity varied widely across the TP (Figure 7). Five meteorological stations (5.5% of the total) with the highest percentage of spring rainfall erosivity were concentrated in the Kunlun Mountains on the Tibetan Plateau near the Pamir Plateau and in the Nu River basin in the Eastern Himalaya. In particular, the erosivity of spring rainfall accounted for more than 50% of Pishan station, Kashgar station, and Zayu station, and Pishan station was as high as 79%. (Figure 7a). Nearly 92% of the meteorological stations (total 84) had the highest percentage of summer rainfall erosivity. The largest was Shiquanhe station, which surprisingly had 94.28% of the annual rainfall erosivity (Figure 7b). There was only one meteorological station with the highest percentage of fall rainfall erosivity, with three stations accounting for more than 30%, namely Nyalam, Burang, and Keriya station (Figure 7c). Winter was the season with the lowest percentage of rainfall erosivity, all stations had less than 30% of rainfall erosivity (Figure 7d). Summer and autumn were the most erosive seasons. It is worth noting that rainfall erosivity was generally higher across the plateau in summer, particularly in the southeastern part of the Tibetan Plateau, whereas rainfall erosivity in autumn and winter was still higher proportion in the south-western part of the plateau near the Himalayas. Thus, extra caution will be needed to prevent aggravation of soil erosion in this region.

### 3.4. Relationship between Rainfall Erosivity and ENSO

#### 3.4.1. Influence of ENSO on Rainfall Erosivity in the Different Regions of TP

The El Niño and La Niña events are the ENSO cycles of the warm and cold periods, respectively. Statistics on the rainfall erosivity in Region I (arid zone) and Region II (humid zone) of the TP and whole plateau during the El Niño (ENSO warm event) and La Niña (ENSO cold event) periods are presented in Table 2.The occurrence and duration of El Niño event and La Niña event were determined based on ONI.

In terms of the degree of influence of cold and warm events on the rainfall erosivity, the average monthly rainfall erosivity for the El Niño event was slightly lower than the La Niña event across the TP, but the average of both events was less than the monthly rainfall erosivity for the period 1971–2020. During the El Niño event, the maximum monthly average rainfall erosivity was 225.18 MJ·mm·ha^−1^·h^−1^ and the minimum value was 120.58 MJ·mm·ha^−1^·h^−1^, with an extreme value ratio of 1.87; during the La Niña event, the maximum monthly average rainfall erosivity was 486.03 MJ·mm·ha^−1^·h^−1^ and the minimum value was 101.67 MJ·mm·ha^−1^·h^−1^, with an extreme value ratio of 4.78; thus, higher variability occurred during the La Niña event. In terms of the presence or absence of ENSO events, the average rainfall erosivity during the ENSO and Non-ENSO periods were 192.67 MJ·mm·ha^−1^·h^−1^ and 213.77 MJ·mm·ha^−1^·h^−1^, respectively. It was evident that the average monthly rainfall erosivity during the non-ENSO period was not only greater than that during the ENSO period, but also greater than the total average monthly rainfall erosivity for the whole study period.

The impact of ENSO events on the monthly mean rainfall erosivity in different regions was notably dissimilar. For Region I, the average monthly rainfall erosivity for the El Niño and La Niña events were 147.17 MJ·mm·ha^−1^·h^−1^ and 173.85 MJ·mm·ha^−1^·h^−1^, respectively. This was higher than the average monthly rainfall erosivity for this region for 1971–2020. Moreover, we found that when El Niño events or La Niña events occurred, there was a significant increase in rainfall erosivity in Region I relative to the Non-ENSO period, but La Niña events had a greater impact on the monthly average rainfall erosivity, compared with El Niño events; For Region II, the average monthly rainfall erosivity for the El Niño and La Niña events were 208.93 MJ·mm·ha^−1^·h^−1^ and 210.13 MJ·mm·ha^−1^·h^−1^, respectively, with the El Niño event slightly lower than the La Niña event. There was a modest gap between the two events. However, compared with Region I, the direction of influence of ENSO in Region II was in the opposite direction. In other words, when ENSO occurs, the average monthly rainfall erosivity in this region decreased more significantly than the average for the study period. Due to the difference in the magnitude of rainfall erosivity on the Tibetan Plateau during the ENSO period and the non-ENSO period, under the premise that other contributing factors was fixed, rainfall erosivity was stronger during the non-ENSO period and soil erosion concerns and soil conservation measures should be strengthened during this period. Considering the obvious spatial heterogeneity of the impact of ENSO on the Tibetan Plateau, the emphasis should be on erosion in the north-west during El Niño or La Niña events, especially during the La Niña event when control measures should be enhanced.

#### 3.4.2. Correlation between Rainfall Erosivity and Multivariate ENSO Index

**Figure 8:**
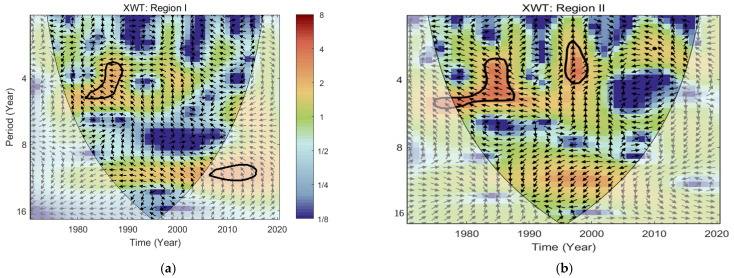
Cross wavelet transforms (XWTs) for annual rainfall erosivity and multivariate ENSO Index (MEI) in Region I (**a**) and Region II (**b**). NOTE: The thick black outline indicates the 95% significance level against red noise, the white translucent area indicates the cone of influence, and the color bar indicates the magnitude of the XWT cross spectral power. That is, red is strong and blue is weak. The arrows (vectors) designate the phase difference between rainfall erosivity and MEI. Where the left arrow indicates the opposite phase relationship between the rainfall erosivity and MEI and vice versa. The north-pointing arrow indicates that the peak rainfall erosivity are lower than the peak MEI.

**Figure 9:**
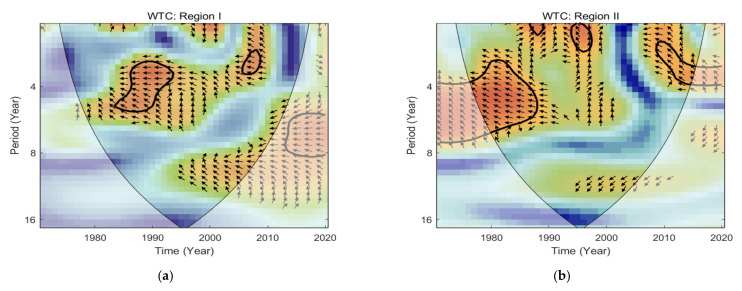
Wavelet transform coherence (WTC) for annual rainfall erosivity and multivariate ENSO Index (MEI) in Region I (**a**) and Region II (**b**). NOTE: The thick black outline indicates the 95% significance level, the white translucent area indicates the cone of influence, and the color bar indicates the significance level of the Monte Carlo test. That is, red means strong correction and blue is weak. where the left arrow indicates the opposite phase relationship between the two-time series, vice versa.

To examine the extent and impact of ENSO on rainfall erosivity, an XWT and WTC analysis were conducted on the time series of rainfall erosivity and MEI index in different regions of the Tibetan Plateau from 1971 to 2020, revealing the periodicity characteristics of both. As shown in Figure 8, in the time-frequency space domain of Region I, it is obvious that there was 3–5 years of high-energy resonance cycle between rainfall erosivity and the MEI index for the period of 1981–1988, during which there was a negative correlation between both time series. In the Region II power spectrum, there were two significant high-energy domains, specifically a 3–5 years resonance cycle from 1981 to 1988 was similar to that of Region I, indicating a consistent ENSO effect across the plateau during this period, but the intensity of the Region II resonance cycle was higher. The other was that there was a 2–5 years resonance cycle of rainfall erosivity and the MEI index from 1995 to 1999, and the mean phase angle was nearly 90° vertically upwards, indicating that the rainfall erosivity change was later than the MEI index. In other words, rainfall erosivity had a lag compared with ENSO over the same period. As shown in Figure 9, in the Region I WTC power spectrum, there were negative phase cycles of 3–5 years and 2–3 years in 1985–1992 and 2006–2009, respectively, indicating a negative correlation between rainfall erosivity and the MEI index during this period. Regarding Region II, there were negative phase cycles of 3–7 years from 1977 to 1988, 1–3 years from 1994 to 1998 and 2–4 years from 2007 to 2013, indicating a negative correlation between rainfall erosivity and the MEI index during these periods. While positive phase cycles of 1–2 years from 1987 to 1989 indicate a positive correlation between rainfall erosivity and the MEI index during this period.

## 4. Discussion

This study revealed the spatial and temporal characteristics of rainfall erosivity on the TP from 1971 to 2020 and their relationship with the ENSO Index. The results showed that the average annual rainfall erosivity on the TP since 1971 was 1071.42 MJ·mm·ha^−1^·h^−1^. According to previous studies, this value is higher than in northwestern China but lower than in southeastern China [74], and the overall degree of erosion is light, approximately 0.5 times the global average [91]. Upward trends are shown for rainfall erosivity during 1971 to 2020. Gu et al. also found an increasing trend in rainfall erosivity from 1981 to 2015 in the Tibet Autonomous Region (TAR) [60], and Wang et al. found a same uptrend in rainfall-runoff erosivity from 1961 to 2012 in Sanjiangyuan region, Qinghai Province [53], which is consistent with this study. Fan et al. [61] used TRMM 3B42 data to assess the spatial and temporal variability of rainfall erosivity in the TAR from 2000 to 2010 and found that the average rainfall erosivity was 768 MJ·mm·ha^−1^·h^−1^, which is lower than the results of this study, probably due to the different extent of the study area and the accuracy of the data. Moreover, the reasons for the trend of increasing annual rainfall erosivity but significant decreasing rainfall erosivity in spring and autumn may be related to the variation in rainfall on the TP [92]. Previous studies have shown that since 1961, the Tibetan Plateau is gradually warming and humidifying [93], which may contribute to an increase in rainfall erosivity. Meanwhile, while changes in the westerly circulation lead to a reduction in rainfall in spring and autumn which in turn affects rainfall erosivity [94].

**Figure 10:**
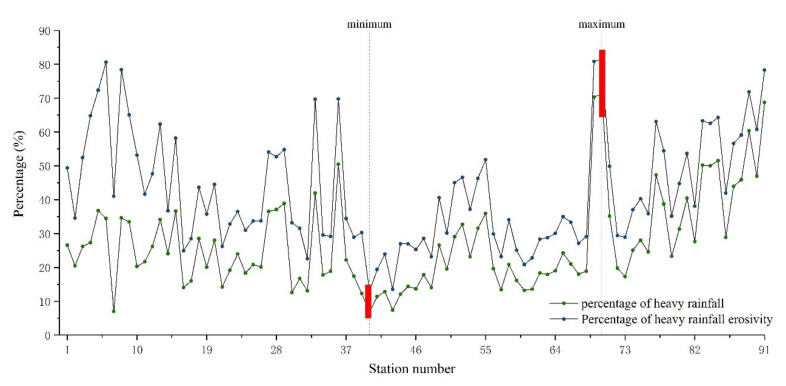
Comparison of the proportion of heavy rain (daily rainfall ≥ 25 mm) and rainfall erosivity for 91 meteorological stations on the TP from 1971 to 2020. Note: the designation of station numbers is shown in Table S3; and the percentage of heavy rainfall represents the proportion of heavy rain amount to total annual precipitation; percentage of heavy rainfall erosivity represents the proportion of heavy rainfall erosivity to annual rainfall erosivity.

In the previous section, this work indicated rainfall erosivity on the TP varied greatly not only seasonally but also monthly. This may be caused by its complicated geography and dominant atmospheric circulation conditions [95]. The plateau spans a wide range of latitudes and longitudes and has a variety of climate types. It is also at the crossroads of monsoon and non-monsoon zones, and is influenced by the prevailing westerly winds, the South Asian monsoon and the East Asian monsoon circulation, resulting in an uneven spatial and temporal distribution of rainfall. Besides, according to previous reports, the amount and intensity of rainfall are the main factors affecting the rainfall erosivity [96]. The spatial and temporal variability of rainfall at various magnitudes will certainly contribute to soil erosion by water at different times and regions to different degrees [97,98]. The average heavy rainfall and average heavy rainfall erosivity for each station from 1971 to 2020 are presented in Table S3. It can be seen from Table S3 that the average heavy rainfall (≥25 mm) of 91 meteorological stations in the plateau was 33.05 mm, and the average rainfall erosivity was 243.35 MJ·mm·ha^−1^·h^−1^. The absolute amount of erosivity caused by heavy rainfall on the TP is low compared with the eastern coastal areas of China, mainly because the total rainfall erosivity in the plateau is much lower than those in the east [35,99,100]. Furthermore, the comparison of the proportion of heavy rain and rainfall erosivity found that the average heavy rainfall on the TP accounted for only 27.02% of the total rainfall, but the rainfall erosivity caused by heavy rainfall occupied by 43.3% of the total rainfall erosivity (Figure 10). In addition, the change in the percentage of heavy rainfall and heavy rainfall erosivity has a high consistency on the TP. In case of heavy rainfall, it can be easily seen that rain erosivity becomes more intense. The maximum values of heavy rainfall and heavy rainfall erosivity were 71.26% and 81.46%, respectively, both of which occurred at Dujiangyan station, and the minimum values of 6.77% and 8.15%, respectively, which occurred at Zhidoi station. Studies have revealed that rainfall is the most important climatic factor contributing to soil erosion, and in particular, heavy rainfall (≥25 mm) is one of the main factors affecting the rainfall erosivity [101]. In general, the higher the intensity of the heavy rainfall, the greater the amount of soil erosion. The frequency of extreme rainfall events is growing as a result of global climate change [102,103], Future research should pay more attention to high intensity rainfall and soil erosion.

The spatial distribution of rainfall erosivity on the TP decreased roughly from south-east to north-west, with significant spatial heterogeneity. Previous studies have shown that mountain tectonics and topographic gradients are essential factors influencing precipitation [104]. The high rainfall erosivity in the south-east is largely attributed to the roughly north-south alignment of the Hengduan Mountains and the gradual rise in elevation from south to north, which is a natural water vapor corridor and facilitates the deeper uplift of monsoon air masses and the formation of rainfall. The north-west of the plateau, on the other hand, is located in an inland region, with high altitude and low temperatures. It is extremely hard for the monsoon to reach it, and rainfall is minimal throughout the year, resulting in lower rainfall erosivity. Besides, in the southern part of the plateau, although it is on the leeward slopes of the Himalayas, where water vapor is not easily accessible, the Yarlung Tsangpo valley is at a relatively low altitude and has locally better hydrothermal conditions, resulting in a higher rainfall erosivity in the region. In addition, the role of anthropogenic activities should not be neglected. Nearly 38.8% of the grasslands on the TP have been degraded [105], and the degradation of meadows caused by overgrazing is a serious environmental and ecological problem. Consequently, soil erosion caused by the contradiction between people and land may exacerbate the rainfall erosivity. 

Clarifying the temporal and spatial patterns of rainfall erosivity on the TP over the past 50 years is of great significance for soil conservation and future land use planning. This paper indicates that the tendency of increasing rainfall erosivity was identified in the southeastern part of the TP. Published studies have shown that the Hengduan Mountains region has the highest soil erosion modulus of the TP [106]. Furthermore, the alternation of steep slopes and deep ravines, the superimposed effect of complex topography and intensified rainfall erosivity may further magnify soil erosion in this region. The Yarlung Tsangpo Valley and the Hehuang Valley are areas of intensive human activity on the TP, and are also major wheat and barley cultivation areas, with crops mostly grown on the slopes of the valleys [107]. Although rainfall erosivity has not shown a sharp rise over the past 50 years in these origins, the region’s originally high rainfall erosivity may still result in frequent natural hazards and elevated ecological risks. The deployment of measures against landslides and debris flows should be considered as a priority. In the western and northern parts of the Tibetan Plateau, there is higher variability, although with lower rainfall erosivity. On the one hand, in the context of increased rainfall erosivity across the plateau, it is still possible to damage the low-cover turf. In particular, the interactive effects of human activities such as grazing and soil erosion processes can also exacerbate the ‘black beach’ degradation of plateau grasslands. On the other hand, the north-west has a higher altitude and fragile natural environment where seasonal differences in rainfall erosivity is more likely to cause damage to alpine ecosystems. Therefore, soil erosion management strategies in this region should not be neglected either.

The impact of global-scale climate oscillation regimes on climate change has received widespread attention. This paper used MEI to characterize the global-scale climate oscillation model ENSO in an attempt to explore the relationship between ENSO and rainfall erosivity on the Tibetan Plateau, with a view to providing guidance for collaborative work on climate change and soil conservation. This study found that rainfall erosivity was lower during ENSO than during non-ENSO, and other studies have found the same results [35,36]. ENSO is a major factor influencing temperature and precipitation in China. Some studies have shown that precipitation anomalies can reach up to 30% of the average precipitation during ENSO periods [108]. It is worth noting that rainfall erosivity was higher during the La Niña period than during the El Niño period on the TP. This is not in agreement with previous studies, with results in places such as Fujian in southeastern China [37], but is consistent with studies in Guizhou in southwestern China [36]. The other research indicated that El Niño occurs with a delayed arrival of the southwest monsoon, while the opposite occurs at La Niña, so this may be related to a weakening of the Indian monsoon [109,110]. These findings would explain the difference of rainfall erosivity between the La Niña period and El Niño period. According to the CWT results, a noticeable resonance cycle between rainfall erosivity and MEI was found in different regions of the Tibetan Plateau, but there were also significant differences in cycle duration, direction of action, and intensity. This may be due to the fact that ENSO events themselves present diversities of climatic features at each stage of occurrence, development, maturation and decline [108]. Additionally, it also suggested that global climate anomalies are an important driver of changes in the rainfall erosivity on the TP.

Due to the vast expanses of land and sparse populations as well as the harsh natural conditions, the distribution of weather stations on the Tibetan Plateau is extremely irregular, which may affect the accuracy of the interpolation. Although this study improves the comprehension of the impact of ENSO on rainfall erosivity, it still lacks further explanation from a mechanistic perspective. Furthermore, in light of the known results, it is clear that not only ENSO but also topography, altitude, and microclimate are associated with rainfall erosivity, and detailed knowledge will be necessary for future studies.

## 5. Conclusions

This study carried out an insightful analysis of the spatial, interannual, and seasonal variability of rainfall erosivity on the TP from 1971 to 2020 and its relationship with ENSO. Daily rainfall data from 91 meteorological stations were collected, and the change trend of rainfall erosivity calculated based on a daily rainfall erosivity model were detected at a regional and site-scale using methods such as Mann–Kendall test and Sen’s slope. The potential influence of ENSO on rainfall erosivity was revealed using the continuous wavelet transform method. The main findings were summarized below:

Rainfall erosivity has shown a fluctuating trend of increasing over the past half century. Seasonal and monthly rainfall erosivity showed high heterogeneity, which was greatly related to heavy rainfall. The rainfall erosivity in order was summer > autumn > spring > winter. July was the most erosive month, accounting for 24.19% of the year, while November was the lowest, accounting for only 1.12%. The rainfall erosivity in spring and autumn showed a significant decreasing trend (*p* < 0.05), and in summer it showed an increasing trend but not significant. There was generally an obvious spatial variation in rainfall erosivity on the TP from 1971 to 2020, presenting a roughly spatial pattern of decreasing distribution from southeast to northwest. Annual rainfall erosivity in the south-eastern part of the plateau was mainly characterized by a slow increase, while in the north-western part annual rainfall erosivity was more unstable with mostly no significant trends.

ENSO events had a significant impact on rainfall erosivity on the TP. The rainfall erosivity in the non-ENSO period was higher than that in the ENSO period, and the La Niña event was higher than the El Niño event. It was also found that there was a clear resonance cycle between rainfall erosivity and ENSO in different regions of the plateau, with an average cycle of about 3–5 years in the high energy region, but there were differences in the timing of occurrence, direction of action, and intensity of the cycle. The rainfall erosivity on the TP was relatively large during non-ENSO periods and relatively small during El Niño/La Niña periods. In addition, the response of rainfall erosivity to ENSO was spatially heterogeneous. Rainfall erosivity in the northwest of TP appears to be more serious during the La Niña event and less severe during the El Niño event. It can be concluded that soil erosion may become more intense during the La Niña event in the northwest TP. Therefore, during the La Niña event, soil protection should be enhanced to diminish soil spattering and disturbance.

This study contributes to the understanding of the spatial and temporal variability of annual rainfall erosivity across the entire TP over the last half century and extends the cognition of the possible impact of changes in ENSO characteristics associated with climate change. Uncertainties may be involved due to limited data availability and interpolation bias errors. Future studies should integrate the effects of multiple factors on rainfall erosivity, more carefully relate the effects of climate extremes, and improve the insights from the mechanistic aspects of change.

## Figures and Tables

**Table 1:** Definition and indicators of rainfall erosivity trend patten.

Trend Patten	Definition	Identification Indicators
DE	Rainfall erosivity decreased significantly during the study period	*Z <* −1.96, or 1.96 ≤ *Z <* −0.675 and *SLOPE%* < −0.25%
ST	Rainfall erosivity showed no significant change during the study period	|*Z*| < 0.675, or 0.675 ≤ |*Z*| < 1.96 and |*SLOPE%*| < 0.25%
IN-ST	Rainfall erosivity showed an increasing trend in the early stage, but showed a stable trend in the later stage	0.675 ≤ *Z* < 1.96 and *SLOPE%* > 0.25% and *R_ST_* < 1, or *Z* ≥ 1.96 and *R_ST_* < 1
IN	Rainfall erosivity showed a gradual increase trend during the study period	0.675 < *Z* < 1.96 and *SLOPE%* > 0.25% and *R_ST_* = 1, or *Z* ≥ 1.96 and *R_ST_* = 1

Note: The *Z* value indicates the MK trend detection value, the *SLOPE%* is the trend change rate percentage, and the *R_ST_* refers to the ratio of the rainfall erosivity in the past 3 years to the maximum 3-year moving average. DE: decreasing, ST: stagnating, IN-ST: increasing-stagnating, IN: increasing.

**Table 2:** Average monthly rainfall erosivity in different regions (Region I and Region II) and TP for El Niño and La Niña events from 1971 to 2020. The bottom table provides a summary of the average monthly rainfall erosivity.

No.	Time Internal	Duration Time	Region I Erosivity	Region II Erosivity	TP Erosivity
El Niño events					
1	1972.05–1973.03	11	282.09	233.00	201.92
2	1976.09–1977.02	6	90.57	216.53	198.37
3	1977.09–1978.01	5	114.40	132.34	120.58
4	1979.10–1980.02	5	104.33	209.91	175.40
5	1982.04–1983.06	15	83.32	214.03	174.49
6	1986.09–1988.02	18	315.97	221.63	225.18
7	1991.05–1992.06	14	106.09	256.33	210.08
8	1994.09–1995.03	7	74.89	167.78	142.50
9	1997.05–1998.05	13	144.15	235.53	193.00
10	2002.06–2003.02	9	141.13	251.03	208.63
11	2004.07–2005.02	8	155.69	247.67	133.83
12	2006.09–2007.01	5	84.40	167.47	188.51
13	2009.07–2010.03	9	128.03	217.52	170.04
14	2014.10–2016.04	19	63.75	193.68	204.68
15	2018.09–2019.06	10	318.80	169.55	201.92
La Niña events					
1	1971.01–1972.01	12	105.87	162.81	142.12
2	1973.05–1974.07	15	101.82	327.52	197.69
3	1974.10–1976.04	19	136.34	246.86	234.16
4	1983.09–1984.01	5	170.46	216.68	191.36
5	1984.10–1985.08	11	125.46	251.72	216.24
6	1988.05–1989.05	13	408.05	261.23	486.03
7	1995.08–1996.03	8	117.67	249.63	190.13
8	1998.07–2001.02	32	153.16	264.05	209.01
9	2005.11–2006.03	5	474.19	124.57	142.75
10	2007.06–2008.06	13	155.75	201.97	178.10
11	2008.11–2009.03	5	283.13	123.77	138.83
12	2010.06–2011.05	12	102.80	214.74	185.26
13	2011.07–2012.04	10	195.09	192.46	187.99
14	2016.08–2016.12	5	109.84	251.42	223.47
15	2017.10–2018.04	7	48.87	116.56	101.67
16	2020.08–2020.12	5	93.12	156.05	229.36
Average monthly erosivity El Niño			147.17	208.93	181.94
Average monthly erosivity La Niña			173.85	210.13	203.39
Average monthly erosivity ENSO			160.51	209.53	192.67
Average monthly erosivity non-ENSO			140.48	253.09	213.77
Average monthly erosivity 1971–2020			146.25	233.68	208.12

## Data Availability

All relevant data sets in this study are described in the manuscript.

## Supplementary Materials

The following are available online at https://www.mdpi.com/article/10.3390/ijerph182111054/s1, Figure S1: Rainfall erosivity anomalies from 1971 to 2017, the blue line shows the 5–year moving average curve, Figure S2: Sequential MK test for 50-year rainfall erosivity of TP. Note: UF and UB refer to progressive and retrograde sequences within the sequence, respectively. UF > 0 indicates an increasing trend, UF < 0 indicates a decreasing trend. The mutation year exists at the intersection of UF and UB. The dashed line represents the 95% confidence interval, Figure S3: Seasonal and monthly average rainfall erosivity Mann–Kendall trends of plateau from 1971 to 2020. ** *p* < 0.01; * *p* < 0.05; + *p* < 0.1, Figure S4: Long–term trend pattern of rainfall erosivity at each station from 1971 to 2020. Note: DE: decreasing, ST: stagnating, IN-ST: increasing-stagnating, IN: increasing, Table S1: The basic information of 91 meteorological stations in this study. ‘No’ refers to station number, which is the same as the station number in Figure 1, Table S2: Statistical results of *Z*, *SLOPE*, and *R_s_**_t_* associated with the rainfall erosivity trend classification at each meteorological station on the TP from 1971 to 2020, Table S3: The average heavy rainfall (AHR) and its proportion (HR) and the proportion of average heavy rain erosivity (HRE) of each station from 1971 to 2020.

## Abbreviations

ENSO	El Niño southern oscillation
TP	Tibetan Plateau
ONI	Oceanic Niño Index
MEI	Multivariate ENSO Index
CWT	Continuous wavelet transforms
XWT	Cross Wavelet Transform
WTC	Wavelet Transform Coherence
CV	Coefficient of variation
