# Supplementary material for: Spatiotemporal Variation in Rainfall Erosivity and Correlation with the ENSO on the Tibetan Plateau since 1971
Source: Int J Environ Res Public Health. 2021 Oct 21;18(21):11054. doi: 10.3390/ijerph182111054 (PMC8583552; doi:10.3390/ijerph182111054)
Supplement: Supplementary file 1 [file ijerph-18-11054-s001.zip › ijerph-1364200-supplementary.pdf]

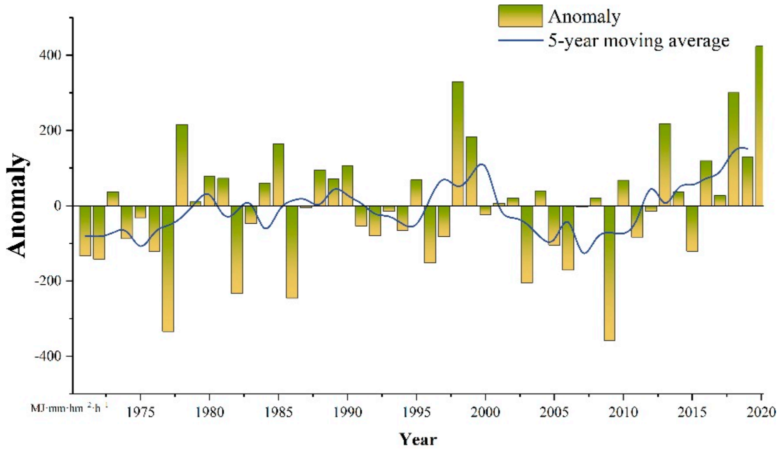

Figure S1. Rainfall erosivity anomalies from 1971 to 2017, the blue line shows the 5–year moving average curve.

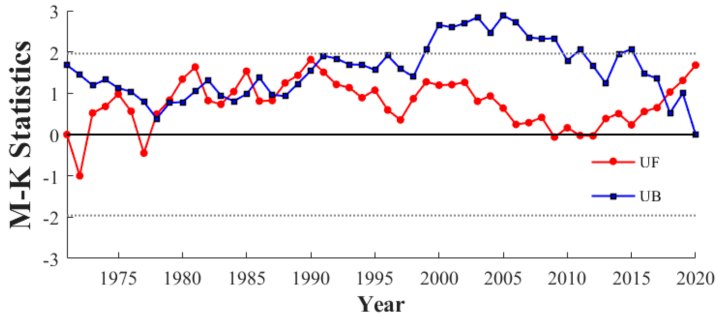

Figure S2. Sequential MK test for 50–year rainfall erosivity of TP. Note: UF and UB refer to progressive and retrograde sequences within the sequence, respectively. UF > 0 indicates an increasing trend, UF < 0 indicates a decreasing trend. The mutation year exists at the intersection of UF and UB. The dashed line represents the 95% confidence interval.

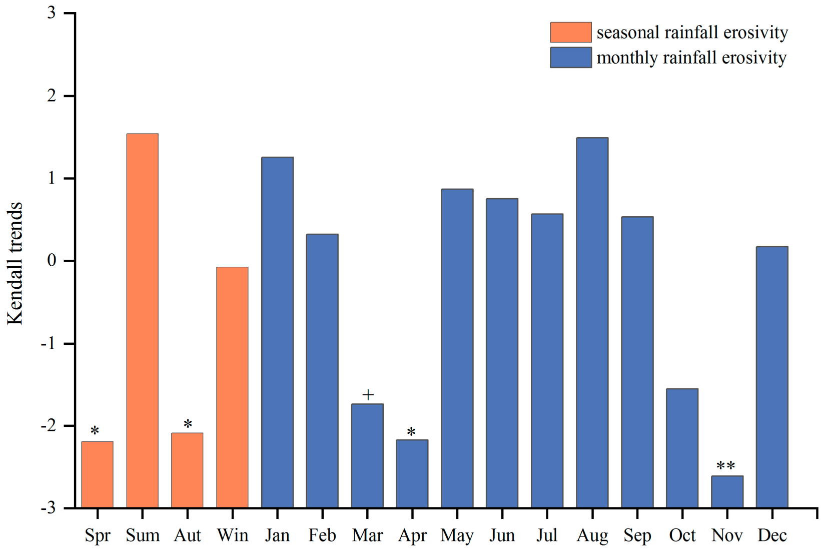

Figure S3. Seasonal and monthly average rainfall erosivity Mann–Kendall trends of plateau from 1971 to 2020. ** $p < 0.01$; * $p < 0.05$; + $p < 0.1$.

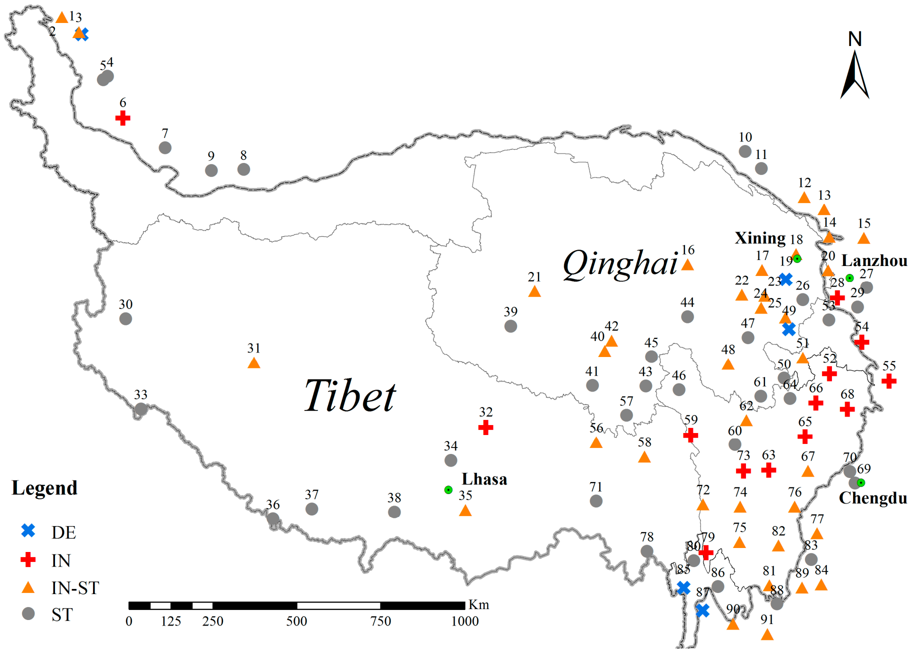

Figure S4. Long–term trend pattern of rainfall erosivity at each station from 1971 to 2020. Note: DE: decreasing, ST: stagnating, IN-ST: increasing-stagnating, IN: increasing.

Table S1. The basic information of 91 meteorological stations in this study. 'No' refers to station number, which is the same as the station number in Figure 1.

No.	Station	Province	Latitude (°N)	Longitude (°E)	Altitude (m)	No.	Station	Province	Latitude (°N)	Longitude (°E)	Altitude (m)
1	Artux	Xinjiang	39.4	76.1	1298.7	47	Golog	Qinghai	34.5	100.3	3719
2	Wuqia	Xinjiang	39.7	75.3	2175.7	48	Darlag	Qinghai	33.8	99.7	3967.5
3	Kashgar	Xinjiang	39.5	76.0	1289.4	49	Henan	Qinghai	34.7	101.6	3500
4	Makit	Xinjiang	38.5	77.4	1178.2	50	Jigzhi	Qinghai	33.4	101.5	3628.5
5	Yarkant	Xinjiang	38.4	77.3	1231.2	51	Maqu	Gansu	34.0	102.1	3471.4
6	Pishan	Xinjiang	37.6	78.3	1375.4	52	Zoige	Sichuan	33.6	103.0	3439.6
7	Hotan	Xinjiang	37.1	79.9	1375	53	Hezuo	Gansu	35.0	102.9	2910
8	Minfeng	Xinjiang	37.1	82.7	1409.5	54	Min	Gansu	34.4	104.0	2315
9	Keriya	Xinjiang	36.9	81.7	1422	55	Wudu	Gansu	33.4	104.9	1079.1
10	Gaotai	Gansu	39.4	99.8	1332.2	56	Dengqen	Tibet	31.4	95.6	3873.1
11	Zhangye	Gansu	38.9	100.4	1482.7	57	Nangqen	Qinghai	32.2	96.5	3643.7
12	Yongchang	Gansu	38.2	102.0	1976.9	58	Chamdo	Tibet	31.1	97.2	3306
13	Wuwei	Gansu	37.9	102.7	1531.5	59	Dege	Sichuan	31.8	98.6	3184
14	Wushaoling	Gansu	37.2	102.9	3045.1	60	Garze	Sichuan	31.6	100.0	3393.5
15	Jingtai	Gansu	37.2	104.1	1630.9	61	Banma	Qinghai	32.9	100.8	3530
16	Dulan	Qinghai	36.3	98.1	3191.1	62	Sertar	Sichuan	32.3	100.3	3893.9
17	Qabqa	Qinghai	36.3	100.6	2835	63	Dawu	Sichuan	31.0	101.1	2957.2
18	Xining	Qinghai	36.7	101.8	2295.2	64	Ngawa	Sichuan	32.9	101.7	3275.1
19	Guizhou	Qinghai	36.0	101.4	2237.1	65	Barkam	Sichuan	31.9	102.2	2664.4
20	Minhe	Qinghai	36.3	102.8	1813.9	66	Hongyuan	Sichuan	32.8	102.6	3491.6
21	Wudaoliang	Qinghai	35.2	93.1	4612.2	67	Xiaojin	Sichuan	31.0	102.3	2369.2
22	Xinghai	Qinghai	35.6	100.0	3323.2	68	Songpan	Sichuan	32.6	103.6	2850.7
23	Guinan	Qinghai	35.6	100.8	3120	69	Wenjiang	Sichuan	30.7	103.8	539.3
24	Tongde	Qinghai	35.3	100.7	3289.4	70	Dujiangyan	Sichuan	31.0	103.7	698.5
25	Zekog	Qinghai	35.0	101.5	3662.8	71	Bome	Tibet	29.9	95.8	2736
26	Tongren	Qinghai	35.5	102.0	2491.4	72	Batang	Sichuan	30.0	99.1	2589.2
27	Yuzhong	Gansu	35.9	104.2	1874.4	73	Xinlong	Sichuan	30.9	100.3	3000
28	Linxia	Gansu	35.6	103.2	1917.2	74	Litang	Sichuan	30.0	100.3	3948.9
29	Lintao	Gansu	35.4	103.8	1893.8	75	Daocheng	Sichuan	29.1	100.3	3727.7
30	Shiquanhe	Tibet	32.5	80.1	4278.6	76	Kangding	Sichuan	30.1	102.0	2615.7
31	Gerze	Tibet	32.1	84.4	4414.9	77	Hanyuan	Sichuan	29.4	102.7	795.9
32	Nagqu	Tibet	31.5	92.1	4507	78	Zayu	Tibet	28.6	97.5	2327.6
33	Burang	Tibet	30.3	81.3	4900	79	Derong	Sichuan	28.7	99.3	2422.9
34	Damxung	Tibet	30.5	91.1	4200	80	Diqing	Yunnan	28.5	98.9	3319
35	Tsetang	Tibet	29.3	91.8	3551.7	81	Muli	Sichuan	27.9	101.3	2426.5
36	Nyalam	Tibet	28.2	86.0	3810	82	Jiulong	Sichuan	29.0	101.5	2987.3
37	Tingri	Tibet	28.6	87.1	4300	83	Yuexi	Sichuan	28.6	102.5	1659.5
38	Gyantse	Tibet	28.9	89.6	4040	84	Zhaojue	Sichuan	28.0	102.8	2132.4
39	Tuotuohe	Qinghai	34.2	92.4	4533.1	85	Gongshan	Yunnan	27.8	98.7	1583.3
40	Zhidoi	Qinghai	33.9	95.6	4179	86	Zhongdian	Yunnan	27.8	99.7	3276.7
41	Zadoi	Qinghai	32.9	95.3	4066.4	87	Weixi	Yunnan	27.2	99.3	2326.1
42	Qumarleb	Qinghai	34.1	95.8	4175	88	Yanyuan	Sichuan	27.4	101.5	2545
43	Yushu	Qinghai	33.0	97.0	3681.2	89	Xichang	Sichuan	27.9	102.3	1590.9
44	Madoi	Qinghai	34.9	98.2	4272.3	90	Lijiang	Yunnan	26.9	100.2	2392.4
45	Qingshuihe	Qinghai	33.8	97.1	4415.4	91	Huaping	Yunnan	26.6	101.3	1244.8
46	Shiqu	Sichuan	33.0	98.1	4200						

Table S2. Statistical results of *Z*, *SLOPE*, and R_{st} associated with the rainfall erosivity trend classification at each meteorological station on the TP from 1971 to 2020.

No.	Station	*Z*	*SLOPE*	R_{st}	No.	Station	*Z*	*SLOPE*	R_{st}
1	Artux	−1.08	−0.87	0.20	47	Golog	0.20	0.50	0.96
2	Wuqia	1.25	1.81	0.64	48	Darlag	1.52	2.11	0.66
3	Kashgar	1.00	0.73	0.95	49	Henan	−0.84	−3.46	0.65
4	Makit	0.45	0.23	0.07	50	Jigzhi	0.20	0.41	0.83
5	Yarkant	−0.24	−0.42	0.23	51	Maqu	1.54	5.65	0.80
6	Pishan	1.05	1.40	1.00	52	Zoige	0.84	4.74	1.00
7	Hotan	−0.23	−0.99	1.00	53	Hezuo	0.65	1.92	0.81
8	Minfeng	0.06	0.21	0.19	54	Min	**2.12** **	8.78	1.00
9	Keriya	0.29	0.28	0.43	55	Wudu	0.70	3.65	1.00
10	Gaotai	0.44	0.19	0.28	56	Dengqen	0.72	2.17	0.89
11	Zhangye	0.20	0.27	0.32	57	Nangqen	−0.12	−0.33	0.63
12	Yongchang	1.82 *	2.10	0.89	58	Chamdo	1.44	3.33	0.68
13	Wuwei	**2.66** ***	3.47	0.97	59	Dege	1.04	3.21	1.00
14	Wushaoling	1.44	3.09	0.83	60	Garze	0.13	0.50	0.99
15	Jingtai	1.49	2.65	0.71	61	Banma	−0.52	−0.86	0.61
16	Dulan	**3.20** ***	2.62	0.64	62	Sertar	1.64	3.51	0.96
17	Qabqa	1.19	1.36	0.83	63	Dawu	1.17	3.58	1.00
18	Xining	1.29	3.01	0.99	64	Ngawa	0.67	1.85	1.00
19	Guizhou	−0.84	−1.15	0.53	65	Barkam	0.95	4.87	1.00
20	Minhe	1.39	3.30	0.90	66	Hongyuan	1.04	4.18	1.00
21	Wudaoliang	**1.65** *	1.71	0.71	67	Xiaojin	1.22	2.63	0.83
22	Xinghai	**1.76** *	3.23	0.82	68	Songpan	1.52	4.46	1.00
23	Guinan	**2.31** **	6.78	0.83	69	Wenjiang	0.47	6.70	1.00
24	Tongde	1.37	3.03	0.62	70	Dujiangyan	0.07	0.70	0.93
25	Zekog	**2.93** ***	6.52	0.72	71	Bome	−0.02	−0.18	0.42
26	Tongren	0.67	1.88	0.94	72	Batang	1.37	3.88	0.46
27	Yuzhong	0.37	1.35	0.99	73	Xinlong	1.59	5.35	1.00
28	Linxia	1.52	6.35	1.00	74	Litang	**1.69** *	5.72	0.50
29	Lintao	−0.62	−2.85	0.57	75	Daocheng	1.66 *	6.10	0.60
30	Shiquanhe	−0.25	−0.24	0.21	76	Kangding	**3.01** ***	11.29	0.82
31	Gerze	**2.92** ***	3.15	0.65	77	Hanyuan	1.54	7.99	0.67
32	Nagqu	1.44	2.86	1.00	78	Zayu	−0.60	−4.20	0.39
33	Burang	−0.40	−0.46	0.09	79	Derong	**2.68** ***	6.64	1.00
34	Damxung	0.33	0.65	0.72	80	Diqing	0.30	1.60	0.48
35	Tsetang	1.20	3.04	0.95	81	Muli	1.57	8.96	0.76
36	Nyalam	−0.57	−5.85	0.37	82	Jiulong	1.22	4.54	0.80
37	Tingri	−0.18	−0.20	0.44	83	Yuexi	0.77	8.66	0.98
38	Gyantse	0.45	0.70	0.64	84	Zhaojue	1.04	8.12	0.69
39	Tuotuohe	0.02	0.04	0.28	85	Gongshan	−1.39	−28.20	0.60
40	Zhidoi	0.73	0.93	0.89	86	Zhongdian	0.22	0.53	0.79
41	Zadoi	0.33	0.63	0.58	87	Weixi	−1.39	−9.72	0.55
42	Qumarleb	1.41	1.72	0.84	88	Yanyuan	−0.02	−0.10	0.90
43	Yushu	−0.30	−0.39	0.78	89	Xichang	1.62	23.59	0.75
44	Madoi	0.30	0.27	0.46	90	Lijiang	0.72	8.20	0.76
45	Qingshuihe	−0.32	−0.65	0.55	91	Huaping	1.17	21.61	0.89
46	Shiqu	0.22	0.35	0.58					

Bold numbers indicate a significant trend and the asterisks designate the confidence interval * 90%, ** 95%, *** 99%. Note: The *Z* indicates the MK trend detection value, the *SLOPE* is the

Sen's slope value of rainfall erosivity changes, and the R_{ST} refers to the ratio of the rainfall erosivity in the past 3 years to the maximum 3-year moving average.

Table S3. The average heavy rainfall (AHR) and its proportion (HR) and the proportion of average heavy rain erosivity (HRE) of each station from 1971 to 2020.

No.	Station	HR (mm)	HRE ($MJ \cdot mm \cdot ha^{-1} \cdot h^{-1}$)	No.	Station	HR (mm)	HRE ($MJ \cdot mm \cdot ha^{-1} \cdot h^{-1}$)
1	Artux	31.43	185.45	47	Golog	29.32	129.34
2	Wuqia	31.77	152.32	48	Darlag	28.92	125.4
3	Kashgar	31.13	239.11	49	Henan	32.7	171.19
4	Makit	33.84	449.9	50	Jigzhi	30.56	143.34
5	Yarkant	33.82	344.29	51	Maqu	34.68	192.09
6	Pishan	33.56	692.46	52	Zoige	32.43	168.95
7	Hotan	27.7	3722.51	53	Hezuo	33.07	176.35
8	Minfeng	32.94	795.06	54	Min	33.56	180.93
9	Keriya	35.14	318.75	55	Wudu	35.44	205.62
10	Gaotai	33.76	330.86	56	Dengqen	30.12	139.41
11	Zhangye	29.4	162.58	57	Nangqen	30.32	138.12
12	Yongchang	32.08	173.37	58	Chamdo	31.54	152.9
13	Wuwei	37.12	246.99	59	Dege	29.45	131.26
14	Wushaoling	30.19	138.5	60	Garze	28.75	124.38
15	Jingtai	34.83	197.66	61	Banma	30.72	142.41
16	Dulan	29.54	134.58	62	Sertar	30.09	140
17	Qabqa	31.9	156.23	63	Dawu	30.92	146.62
18	Xining	33.05	170.02	64	Ngawa	31.01	148.56
19	Guizhou	31.25	154.48	65	Barkam	30.85	150.5
20	Minhe	34.37	190.82	66	Hongyuan	31.56	156.08
21	Wudaoliang	29.91	136.52	67	Xiaojin	29.21	129.23
22	Xinghai	31.7	153.38	68	Songpan	30.44	142
23	Guinan	31.44	153	69	Wenjiang	51.23	500.45
24	Tongde	31.36	153.6	70	Dujiangyan	51.45	512.46
25	Zekog	31.94	156.91	71	Bome	34.22	197.21
26	Tongren	32.03	163.28	72	Batang	30.45	141.98
27	Yuzhong	35.66	210.26	73	Xinlong	30.86	154.56
28	Linxia	34.99	203	74	Litang	32.24	163.7
29	Lintao	34.94	207.82	75	Daocheng	31.24	154.04
30	Shiquanhe	28.57	257.95	76	Kangding	30.78	149.39
31	Gerze	31.84	166.11	77	Hanyuan	38.84	267.52
32	Nagqu	29.79	134.31	78	Zayu	37.09	233.94
33	Burang	38.66	276.68	79	Derong	31.01	146.88
34	Damxung	31.33	149.03	80	Diqing	32.64	170.43
35	Tsetang	29.76	133.85	81	Muli	34.18	108.73
36	Nyalam	45.79	375.94	82	Jiulong	30.45	149.47
37	Tingri	31.83	156.31	83	Yuexi	37.03	245.11
38	Gyantse	29.68	134.18	84	Zhaojue	36.34	231.52
39	Tuotuohe	34.91	213.06	85	Gongshan	37.99	258.23
40	Zhidoi	27.96	116.06	86	Zhongdian	32.76	171.3
41	Zadoi	29	124.27	87	Weixi	34.66	206.75
42	Qumarleb	30.71	142.08	88	Yanyuan	36.06	222.45
43	Yushu	29.63	131.44	89	Xichang	41.16	311.65
44	Madoi	31.72	165.94	90	Lijiang	37.53	245.24

45	Qingshuihe	31.6	156.27	91	Huaping	44.82	386.77
46	Shiqu	31.29	153.53		Study area	33.05	243.35
